# Experience With Biobrane: Uses and Caveats for Success

**Published:** 2009-06-26

**Authors:** John E. Greenwood, Jill Clausen, Sheila Kavanagh

**Affiliations:** Burns Unit, Royal Adelaide Hospital, North Terrace, Adelaide 5000, South Australia

## Abstract

**Objective**: To present some of our experience with Biobrane™ (a total of 703 patients in 7 years) in a range of uses in burn practice and to illustrate the caveats that we have found applicable in maintaining our success with this versatile material. **Methods**: Retrospective analysis of theatre records, medical notes, and photography database to categorize our experience with Biobrane. Thorough assessment of our surgical and nursing protocols (both literature and experience influenced) to identify caveats for successful use. **Results**: Pivotal steps are revealed in wound selection, wound preparation, material application, dressing, and subsequent nursing care that have led to overwhelming success in definitive management of superficial partial thickness to middermal burns (ensuring pain relief, allowing early mobilization, tolerance of dressing changes and therapy, and earlier hospital discharge). Its many uses in a broad range of common burn situations are demonstrated and tips provided to achieve best outcome. **Conclusions**: Biobrane is not a panacea but it is extremely versatile. The different potential uses have learning curves, and suggestions are provided to flatten these.

Biobrane™ was first introduced for the treatment of burn wounds and donor sites in 1979.^1^ It is a biosynthetic semipermeable membrane designed to temporarily perform the functions of lost epidermis until reepithelialization. It is bilaminate material (Fig 1), consisting of a woven nonbiodegradable nylon mesh, the superficial (outer) surface of which has a silastic coating (a mechanical barrier to vapor loss and bacterial ingress). Peptides derived from porcine type I collagen are bonded to all exposed nylon and silicone surfaces. The material bonds firmly to the adequately prepared bed of an appropriate burn (superficial partial thickness to middermal injury) until spontaneous detachment by reepithelialization. It is a lightweight material and is packaged and stored as dry sheets or gloves and has a long shelf-life. Our experience with Biobrane (a total of 703 patients in 7 years) reveals many other applications in burns practice, with some patients receiving Biobrane for more than 1 application.

## USES OF BIOBRANE

“Standard” Biobrane (15/3 denier nylon fibers) has been used exclusively for the following applications. The number in parentheses represents the number of cases in which Biobrane has been used for each application:

- Definitive treatment of superficial partial thickness and mid-dermal burns (484 as sole treatment plus 86 where grafting was performed in another area during the same operation)
- “Trial of life” (retrospectively indistinguishable from graft reduction, see below)
- Graft Reduction (plus “trial of life”—65)
- Salvage in the elderly (30)
- Graft holding in difficult sites (35)
- Graft holding in deep areas surrounded by superficial burn (33)
- Postdermabrasion dressing (9)
- Retention of cell suspensions (65)
- Temporizing dressing postexcision of full-thickness burn (18)

### Definitive treatment

**Figure 2:**
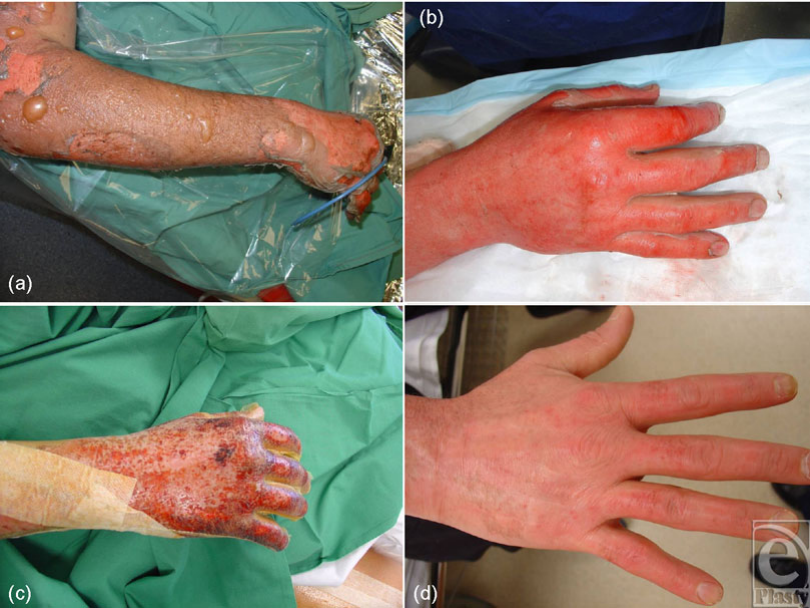
Definitive management of superficial partial thickness burns with Biobrane. An extensively blistered burn wound (a) is meticulously cleaned (b) a Biobrane glove has been applied (c) excellent healing occurs by day 9 (d).

Failure of Biobrane, when it is used for the definitive treatment of superficial partial thickness to middermal burns, is rare in my practice. Success, however, is governed by a number of caveats that I have found to be unbreakable. Loss of Biobrane is chiefly due to nonadherence followed by infection. It cannot be used as definitive treatment in deeper (nonexuding) burns; these require excision and split skin grafting. The primary caveat, therefore, is burn selection. Obvious exudation, or the presence of blisters (popped or intact), indicates an intact subdermal circulation (Fig 2a). An exuding wound bed often cannot be fully appraised without the second caveat—meticulous wound cleaning and preparation (Fig 2b). This has seldom been possible without general anesthesia since it involves aggressive cleaning to remove blister roofs/fragments, other nonviable tissue, and surface contaminants such as dirt and soot. This process also reduces surface contamination by organisms that might have populated the wound during the “cooling” process (clean running water not always available) or during the burning process (wound infection seems more common in burns caused by “organic” agents such as cooking oils, stocks and water, sauces, petrol, etc). Following cleaning, the burn wound and an area of surrounding unburned skin (up to 10 cm from the burn edge) is shaved of hair. Hair might “lay down” during dressing application, but springs up later—the dressing detaches, and air pockets develop, which encourage bacterial ingress. Burns involving hands or feet need nail trimming and scrubbing to reduce these as a source of bacteria. These preparatory stages occur before “scrubbing up.” After full surgical scrub, the skin is prepared with full-strength Betadine™ and sterile drapes are applied around the surgical field. Residual Betadine is washed off with saline—it can be an irritant under Biobrane, especially at the unburned margin. Tincture of Benzoin applied around the burn helps both Biobrane and adhesive tapes to adhere. Biobrane is applied (silastic shiny side out) and anchored at one margin with a 4-cm wide strip of sterile Hypafix™. The opposite side is then similarly anchored under slight tension to eradicate any wrinkling of the Biobrane. The other 2 sides are then anchored; it is useful to pick up the edge of the Biobrane with the tape that can then be pulled outward, affording required tension and ensuring excellent apposition of Biobrane and burn. On the limbs, Biobrane overdressed with full-strength Betadine-soaked Nufold gauze dries out, becoming stiff, uncomfortable, and resistant to movement/exercise. Weak (1 in 10) Betadine (100-mL Betadine made up to 1 L with sterile 0.9% saline) remains soft and conforming. To reduce dressing bulk, the gauze is held on directly with crêpe bandage, reducing heat and moisture at the wound site and limiting maceration. On the trunk, Biobrane is overdressed with Acticoat™ and Hypafix—the treated back lies on an Exudry™ while the treated front is covered by Cutilin™. Postoperatively the treated part is elevated. Outer dressings are replaced daily. Certain structures, the nipples and the umbilicus in particular, should not be covered with Biobrane. A hole is cut out of the Biobrane to allow these structures to be externalized. Betadine ointment is applied to reduce their potential as bacterial sources. The Biobrane glove has reduced surgical time after preparation for hand burns from 30–40 minutes each down to 5–10 minutes each (Fig 2c). The fingers are wrapped individually, the palm bolstered and a “boxing glove” created (with MCPJs, PIPs, and DIPS in flexion) with a crêpe bandage. This maximally stretches the dorsal hand burns in the knowledge that it is easier to regain finger extension than finger flexion (Fig 2d).

### “Trial of Life”

Even after meticulous preparation, some burns appear to sit on the boundary between mid- and deep dermal; sometimes the boundary itself is indistinct. These burns sluggishly refill and the exudation from them is minimal; but if they might be even slightly capable of healing spontaneously, they receive Biobrane as a “Trial of Life.” Preparation of the burn wound is exactly the same as for definitive treatment, but the patient is counseled that success is not guaranteed and excision and grafting may be required if Biobrane fails over the deeper areas.

### Graft reduction

**Figure 3:**
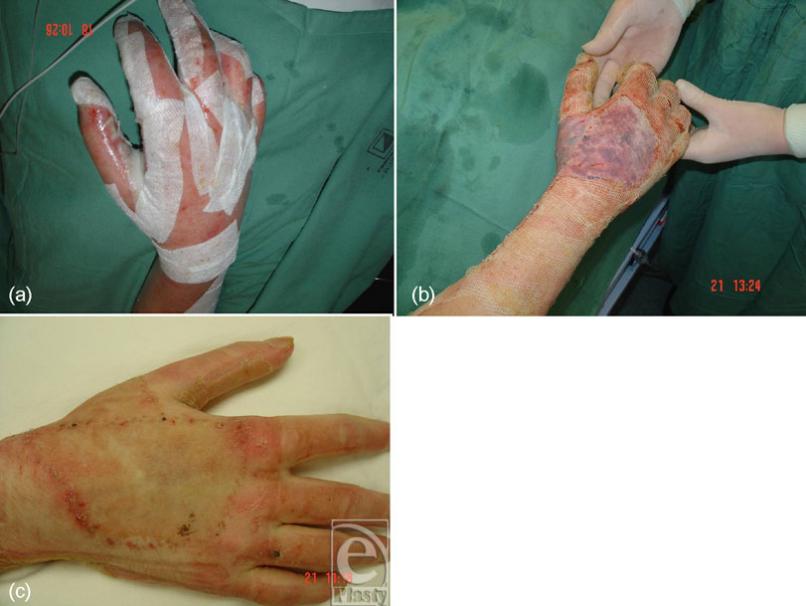
Graft reduction; the superficial burn components heal with Biobrane while the deeper area demarcates allowing accurate grafting with minimal sacrifice of viable tissue.

In certain circumstances, a heterogeneous burn with an equivocal edge to the deeper areas receives Biobrane, according to definitive treatment protocols (Fig 3a). In these cases, there is no doubt that these areas need to be excised and grafted but Biobrane is applied to allow the more superficial burns around them to heal and the edge to demarcate clearly. At excision, only nonviable tissue is excised and the least amount of graft possible is necessary (Figs 3b and 3c).

### Salvage in the elderly

**Figure 4:**
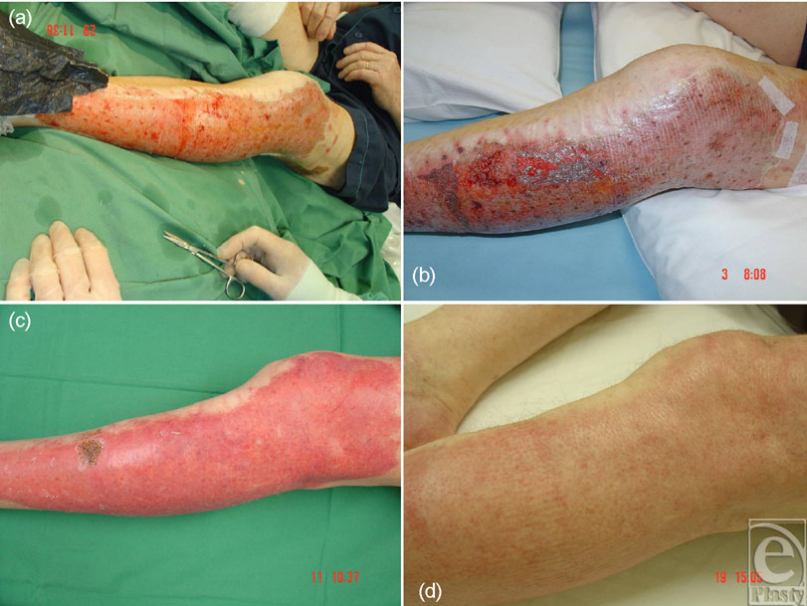
Salvage in the elderly; initially appearing superficial, this burn in a 78-year-old struggled to heal spontaneously but resulted in the best outcome.

The progressive thinning of the dermis and reduction in the density of adenexal structures in the elderly does not enable accurate prediction of spontaneous healing in burns that initially appear to be superficial partial thickness/mid-dermal in depth. This fact often catches out primary caregivers who treat with silver sulfadiazine (SSD) cream or Acticoat and discover, at review a few days later, that the burn has deepened, necessitating excision and grafting. Biobrane applied within 24 hours of the injury (and usually within a couple of hours of immediate post–burn admission) can salvage these burns (Figs 4a and 4b). Whether due to decontamination and preparation of the burn or the application of Biobrane, this procedure is more frequently successful than not (Figs 4c and 4d). Since the elderly cope poorly with a split-skin graft donor site (in terms of both healing and mobilization), this salvage considerably reduces the morbidity of their injury. Failure is usually obvious within 3 days and definitive excision and grafting can then be performed.

### Autograft holding in difficult sites

In major burn injury >50% total body surface area (TBSA), where the back is involved and the patient initially managed on the intensive care unit (ICU), shear causes problems with split skin grafts on the back (despite Therakair™ mattresses). Shear often results from pulling the patient up the bed when being nursed at 30°–45° to reduce facial swelling. When Biobrane is applied over meshed split skin graft, it adheres firmly to the interstices; effectively “spot-welding” it into place (Fig 7d). Acticoat and Exudry outer dressings are applied, and Biobrane is examined daily. The graft is normally well taken by day 7 when SSD cream is applied (which loosens the fibrous clots holding the Biobrane) and the material can be removed easily on day 8. This process does not appear to delay healing of the interstices at all. Biobrane also markedly assists in the holding of widely meshed skin grafts over vascularized Integra™ during the serial delamination/grafting cycle in major burn injury.

### Autograft holding in deep areas surrounded by superficial burn

**Figure 5:**
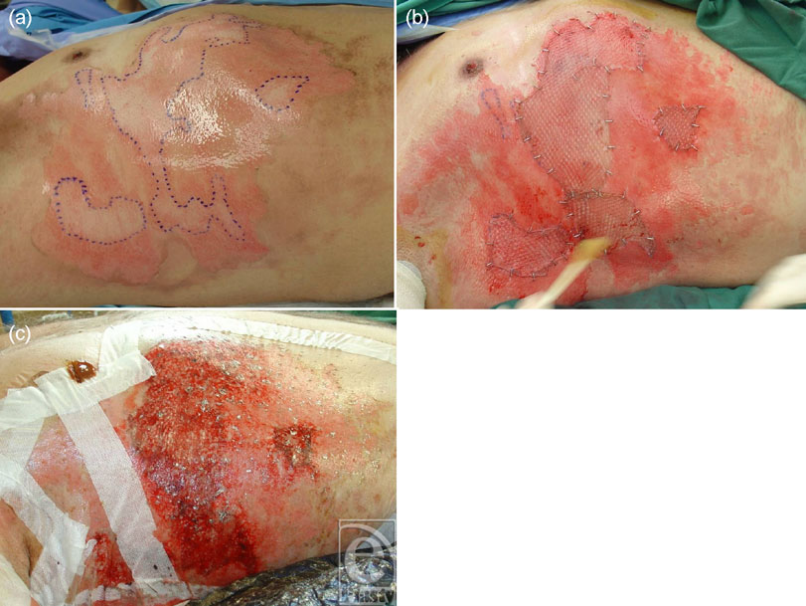
Once the deeper areas have been excised, Biobrane holds the graft and allows the superficial surrounds to heal spontaneously. Note that the nipple has been excluded from the Biobrane and has been treated with Betadine ointment.

In cases of heterogeneous burns where the burn requiring excision is well demarcated immediately (Fig 5a), the deeper areas are excised and grafted (Fig 5b). If these areas are within a patch of superficial burn, Biobrane is applied over the whole wound (Fig 5c). The superficial burn is thus definitively treated by the Biobrane and the graft is firmly held in place. When the superficial areas are clinically healed (usually by day 9 or 10), SSD cream is applied as for “graft holding in difficult sites.”

### Postdermabrasion dressing

**Figure 6:**
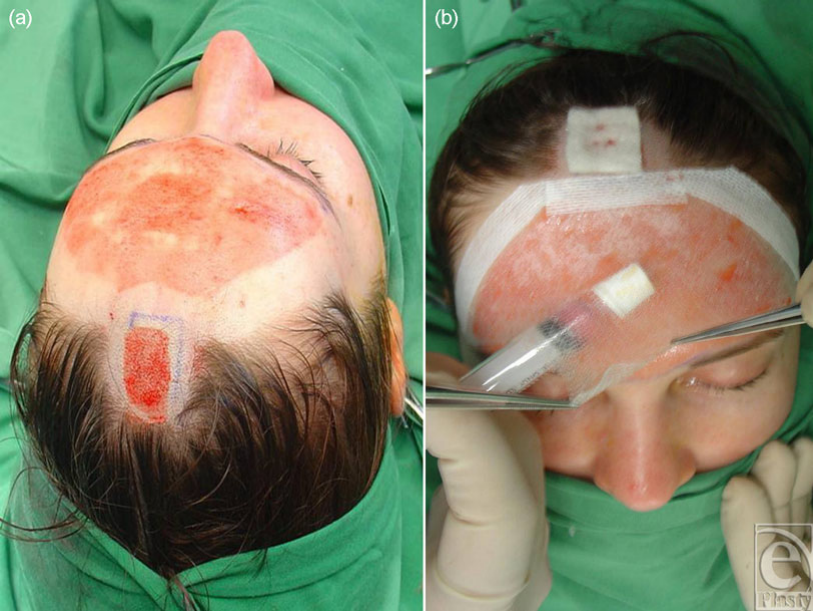
Forehead dermabrasion receives Biobrane, which is also used to retain a cell suspension.

When dermabrasion has been used for burn scar contour correction or repigmentation of burn hypopigmentation (Figs 6a and 6b), Biobrane is often an excellent dressing option, since the dermabraded bed is effectively a superficial partial thickness/middermal wound. This option is possible only if a hemostatic tumescent technique has been employed to facilitate the surgery. In my experience, Biobrane applied to a bleeding bed is held by dense blood clot rather than fine fibrous clot and its subsequent detachment and removal is much more difficult.

### Retention of cell suspensions

**Figure 7:**
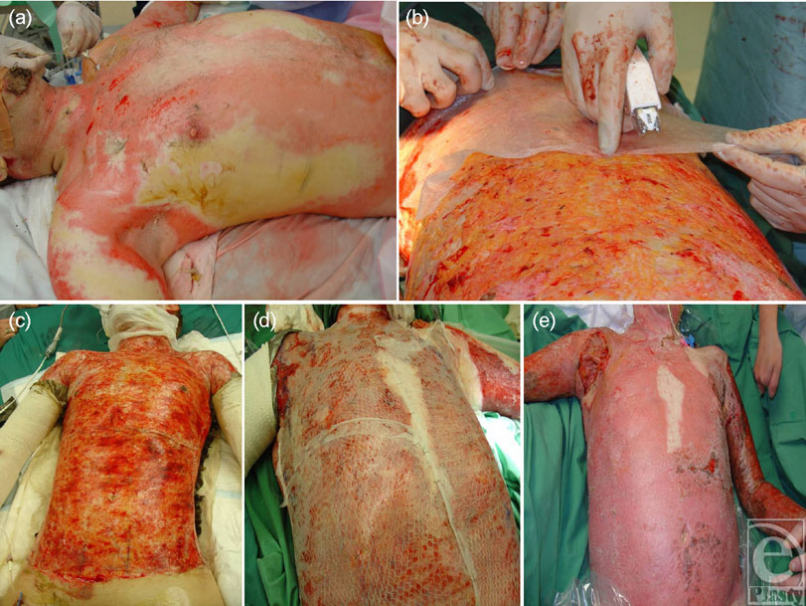
An 80% TBSA burn (a) receives Biobrane initially as a temporizing dressing (b and c) and later to hold meshed graft and cultured cell suspension (d) resulting in healing (e).

When cultured keratinocyte suspensions are applied over meshed split skin graft in our patients with burns >50% TBSA, Biobrane is the retention dressing of choice (Fig 7d). Despite the 1-mm pores present in the film, the silastic layer generally minimizes cell/media loss. This appears to be more effective than other commonly used materials such as Surfasoft™.

### Temporizing dressing postburn excision

The tumescent technique we employ enables the complete excision of all burn eschar (even in >80% TBSA burns; Fig 7a). Temporary closure is then essential until physiological improvement is displayed by the patient. Available split-skin graft or Integra is never applied within the first 48 hours of burn excision in the patient in the ICU. Any physiological deterioration in this time tends to result in the use of inotropes, which shut down the peripheral circulation. This can result in loss of graft and Integra and conversion of skin graft donor sites to full-thickness injuries (not only increasing the size of the wound needing grafting but reducing donor site availability). Following burn excision, therefore, Biobrane is applied to the underlying fat as a temporizing dressing (Figs 7b and 7c). This usually stays in situ for 3 to 6 days until definitive closure can be afforded (most often with Integra although the back is always grafted).

## DISCUSSION

To justify its expense and operative placement under general anesthesia, the use of Biobrane for the definitive management of spontaneously healing burns has to display advantages over alternative, less invasive dressing regimes. The 2 main parameters are the degree of postoperative pain and the length of stay in hospital. A third parameter might be the time to return to work. Pain is extremely important; it dictates how much narcotic analgesic will be required. This, together with the need for physiotherapy and occupational therapy, will dictate how quickly and how completely the patient will regain function. Obviously, the sooner patients can cope with their injury by themselves, the sooner they can be discharged and the sooner they will return to work.

In 2007, we surveyed 60 patients, with an average age of 42.4 years (range, 16–82), who had received Biobrane for definitive treatment of superficial partial thickness to middermal burns. We were interested in their pain scores, measured by Visual Analogue Scale, before and after the application of Biobrane. Overall, the background pain score before application was 6.4, rising with examination. Most also remembered feeling “groggy” due to opiates. After the application of Biobrane, the pain scores fell to an average of 3.8. This fall in pain score is despite 8 patients of the 60 surveyed who complained that pain *increased* after the Biobrane application (presumably due to the aggression of their in-theatre debridement). The average time to return to work was 4.5 weeks, longest in those with hand burns and working in hot environments (foundries, kitchens, etc). Figures from the Burns Round-Table (comparative data for all Burns Services in Australia and New Zealand) show that we enjoy the shortest lengths of stay of any unit. The appropriate use of Biobrane in superficial partial thickness and middermal burns has been pivotal in enabling earlier discharge. Our general experience with Biobrane use tallies well with the experience of its early evaluators in several applications.

Retrospective analysis also shows that Biobrane becomes progressively more effective in reducing length of stay as the burn size increases (Table 1).

A small number of patients skewed the data, spending far longer in hospital than their group peers. Further analysis of demography and burn site revealed that patients stayed in hospital longer if they

– were elderly,

– lived alone or were homeless,

– came from rural locations, and

– had hand burns requiring therapy or burns to other “special areas” (feet, genitalia, breasts, buttocks) making early discharge impossible.

Those patients from 2002 (my first year as a burns specialist) stayed slightly longer than subsequent years; an obvious learning curve. Readers might question such aggressive management of small (<10% TBSA) burns by this method and why this particular course is favored. I am the sole burns surgeon in a State unit with 8 beds and 20 nurses and responsible for a catchment area of 2.4 million square kilometers. In 2008, we accepted 456 admissions and the unit managed more than 2500 outpatient visits. Without the expeditious application of Biobrane, this workload could not be managed without greatly increasing the in- and outpatient dressing burden on the nurses and increasing the length of stay. Additional support for this aggressive approach is provided by Phillips et al.^2^ Their paper reported experience on 851 patients and identified 2 potential areas of Biobrane failure; namely inaccurate assessment of burn depth and application of Biobrane over pretreated wounds. Removal of all nonviable tissue and meticulous cleaning of the burn bed to stimulate exudation eradicates both these concerns. The infection rate (5.8%) reported in that paper is 10-fold greater than our own.

The need for meticulous preparation of superficial burns before the application of Biobrane is not a new concept. Demling^3^ elucidated most of the factors I find important, although he makes no reference to whether his “aggressive debridement” required a general anesthetic. He also highlights that Biobrane will not adhere in deeper wound environments, using the failure of Biobrane in such cases as a diagnostic test similar to my “trial of life” application.

I have been asked on several occasions why I apply Biobrane rather than cadaver allograft as a temporizing dressing after excision of major burn injury. The 2 cadaver banks available to Adelaide are based in Melbourne (Victoria, Australia) and Auckland (New Zealand). Both have faced great difficulty in generating and maintaining stock, and more often than not, requests for large volumes of allograft are not met with supply, because of stock shortage or exhaustion. Cadaver allograft from both sources is expensive and of variable quality. It has a limited shelf life and the Therapeutics Goods Administration will not allow its storage in Adelaide if it is not used immediately. Stock that has displayed “acceptable” contamination with *Staphylococcus aureus* or other potential pathogens is sometimes provided. Distance (Melbourne to Adelaide, 732 km; Auckland to Adelaide, 3750 km) and delay (both banks are at least 1 airplane flight away) compound the supply problem. Many of these issues were raised by Purdue and colleagues^4^ during their prospective study in 1985–1987. This multicenter study failed to show any significant difference between cadaver allograft and Biobrane for this application while awaiting definitive autografting.

Even the use of Biobrane to hold meshed autograft has historical precedent with previous authors having similar success when comparing Biobrane with a range of biological materials including cadaver allograft and porcine xenograft.^1^,^5^

The success of early authors (particularly J. F. Hansbrough^6^ and W. Hansbrough^7^) in making Biobrane work on donor sites has been impossible to emulate. I thought that fastidious donor site preparation with adrenaline tumescence (so that the donor site does not bleed but merely exudes serum) would guarantee success. However, Biobrane seemed consistently to become “cemented” to the donor site with sanguinous clot. Its detachment was almost impossible without anesthetic and removal disturbed reepithelialization. Patients also complained that they found its presence on donor sites to be extremely uncomfortable and outer dressing changes excruciating. In addition, the quality of reepithelialization appears more fragile during reharvesting than my usual hydrocolloid method. These findings were considerably disappointing since success would certainly have been advantageous in major burn injury (reducing dressing time, allowing continuation of the temporizing Biobrane sheets over adjacent donor areas, retaining cultured keratinocyte suspensions for expedition of donor site reepithelialization, etc). Since I have had great success with hydrocolloids in donor site management, Biobrane was discontinued for this application.

Similarly, the use of Biobrane on the face is difficult and disappointing. The elasticity that confers excellent appositional advantage on convex surfaces such as the limbs and trunk causes the material to “tent” over concavities such as the orbits, nasomaxillary, and nasolabial folds. The need to staple or suture the material into these creases removed any advantage over soft paraffin face care. We enjoyed great success with Transcyte™ in face management, especially in the intensive care setting, finding face care to be its only indication; agreeing with Demling and DeSanti.^8^ Removal of Transcyte from the market has prompted a return to simple paraffin face care, although we occasionally use AquacelAg™ in intubated patients.

The adherence of Biobrane is probably due to the conversion of fibrinogen (in the burn exudate) to monomeric fibrin following its exposure to Biobrane's “foreign” fragments of porcine collagen. The material is then held by the fine, fibrous “clot.” This explains its failure to adhere to deeper (nonexuding) burn wounds. The same process occurs when Biobrane is applied to a bleeding wound bed, except the result is a dense blood clot that makes detachment problematic. The fibrovascular ingrowth, which occurs when Biobrane is applied to excised burn beds following deep burn debridement, can be disastrous if ignored. The nylon scaffold is not biodegradable and, once ingrown, requires surgical removal (often setting the wound back). Although I have several indications for applying Biobrane to a deep wound bed (graft holding and temporization of the burn wound in the big burn), strict protocols ensure its timely and easy removal in these situations.^9^

Problems with Biobrane are more frequently a fault of technique rather than material. Biobrane is quite ubiquitous when one remembers what it consists of and realizes what can be expected of it.^10^ Like all burn treatments, it is not a panacea; it is a tool that has specific uses. It has disadvantages also. In Australia, it has only very recently been granted the Therapeutic Goods Administration approval for use, previously restricting those who could use it, requiring annual submission of cases in whom it had been used, plus a letter from the hospital ethics committee supporting its continued use. It is expensive relative to other dressing materials for spontaneously healing burns (especially the gloves). The meticulous wound preparation necessary for success requires a general anesthetic, or Biobrane loss can be expected. Regulation has made it a highly specialised dressing, so discharge of patients treated with Biobrane to rural areas is usually delayed. It cannot be applied on delayed presentation burns. Considerable experience is necessary for use in other roles (ie, graft holding) but experience has been difficult to acquire previously because of regulation.

### Standard nursing management

A robust nursing protocol must exist to monitor the progression of the product and direct nursing intervention, especially when Biobrane is introduced as a new product into a burns service or used on outlying patients from the burns unit (eg, ICU). Biobrane gloves are encased in a “boxing glove” bandage and left intact for 3 days. Biobrane applied for any indication except dermabrasion (where the risk of surface contamination is minimal) is inspected daily for signs of infection or nonadherence. Any air or fluid collections are removed. If Acticoat is used as the outer dressing, this is lifted, Biobrane inspected, and Acticoat placed back in position, only being replaced as per normal use. Once exudate ceases, a dry dressing can be applied. One of the main challenges is to prevent the inadvertent early removal of Biobrane before it has been allowed to fulfill its function. If Hypafix or staples are used to fix the material, they should not be removed prior to the product being well adhered. Trimming of Biobrane occurs as the material becomes dry and opaque, indicating healed burn underneath.

If Biobrane does not adhere, but there is a clean wound underneath, the nurses are instructed not to remove the product. In the case of a localized nonadherent area associated with purulence, only the local area should be deroofed. Swabs from the underlying wound should be taken and sent for microbial culture, the wound cleaned and Acticoat applied. In the case of widespread nonadherence associated with purulence, an urgent medical review is sought. Normal practice in this situation is removal of all loose Biobrane, wound swabs are taken for culture, the wound is cleaned, and Acticoat is applied.

## CONCLUSION

Biobrane is ubiquitous and used in my practice for a range of applications. It is relatively inexpensive, easy to store, apply and fix, and reliable when used according to guidelines. In the definitive management of burns expected to heal spontaneously, failure is very uncommon. Although experience is necessary for the other applications mentioned, use is usually rewarded with a course and result at least comparable with, and usually better than, any alternative (Fig 7e). It is important, however, to know its limitations. Recently, Aubrey Woodroof (the inventor of Biobrane) has developed an *Advanced Wound* Bioengineered Alternative Tissue (*AW*BAT™).^11^ This material employs a similar structure to Biobrane except the nylon filaments are finer (15/2 denier on standard AWBAT), the pores are more regular and frequent with uninterrupted nylon mesh flooring them and the porcine collagen peptides are not cross-linked (providing less steric hindrance in their interaction with burn wound exudate), thus expediting adherence. I look forward to comparing this new material with Biobrane.

## Figures and Tables

**Figure 1:**
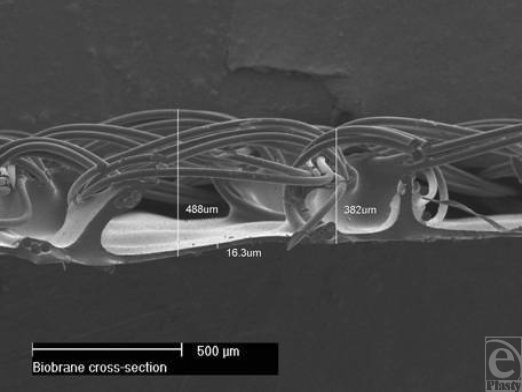
Cross-sectional electron micrograph of Biobrane structure.

**Table 1:** Hospital stay with increasing burn size after Biobrane application

Burn range (sample number)	1%–4% (*n* = 334)	5%–9% (*n* = 72)	10%–19% (*n* = 42)	20%–29% (*n* = 13)	30%–39% (*n* = 8)
Average TBSA	2.52	7.13	12.43	22.54	33.63
Length of stay	4.73	8.07	9.69	11.85	13.13
Days/TBSA	1.88	1.13	0.78	0.53	0.39
